# scRNA-seq reveals the diversity of the developing cardiac cell lineage and molecular players in heart rhythm regulation

**DOI:** 10.1016/j.isci.2024.110083

**Published:** 2024-05-22

**Authors:** Karim Abu Nahia, Agata Sulej, Maciej Migdał, Natalia Ochocka, Richard Ho, Bożena Kamińska, Marcin Zagorski, Cecilia Lanny Winata

**Affiliations:** 1 International Institute of Molecular and Cell Biology, Warsaw, Poland; 2 Laboratory of Molecular Neurobiology, Nencki Institute of Experimental Biology, Warsaw, Poland; 3 Institute of Theoretical Physics and Mark Kac Center for Complex Systems Research, Jagiellonian University, Cracow, Poland; 4 The Njord Centre, Department of Physics, University of Oslo, Oslo, Norway

**Keywords:** Embryology, Integrative aspects of cell biology, Model organism, Transcriptomics

## Abstract

We utilized scRNA-seq to delineate the diversity of cell types in the zebrafish heart. Transcriptome profiling of over 50,000 cells at 48 and 72 hpf defined at least 18 discrete cell lineages of the developing heart. Utilizing well-established gene signatures, we identified a population of cells likely to be the primary pacemaker and characterized the transcriptome profile defining this critical cell type. Two previously uncharacterized genes, *atp1b3b* and *colec10*, were found to be enriched in the sinoatrial cardiomyocytes. CRISPR/Cas9-mediated knockout of these two genes significantly reduced heart rate, implicating their role in cardiac development and conduction. Additionally, we describe other cardiac cell lineages, including the endothelial and neural cells, providing their expression profiles as a resource. Our results established a detailed atlas of the developing heart, providing valuable insights into cellular and molecular mechanisms, and pinpointed potential new players in heart rhythm regulation.

## Highlights

• scRNA-seq analysis revealed diverse cell lineages of the developing zebrafish heart
• Molecular diversity of myocardial subpopulations suggests functional specializations
• Novel role of pacemaker-enriched *atp1b3b* and *colec10* in heart rate and morphogenesis
• Single cell transcriptome profile of various cardiac cell types as valuable resource

## Graphical abstract

**Figure:**
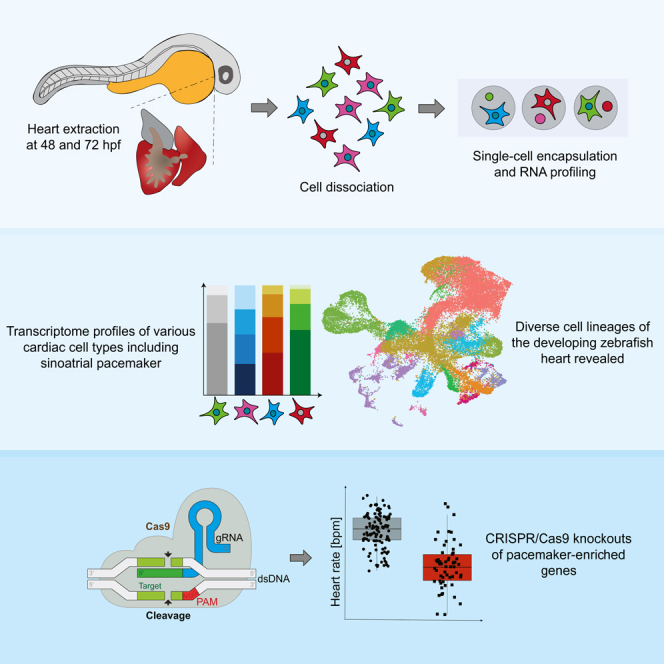

## Introduction

Essential components of the heart ensure its life-sustaining activity.^1^ Specialized cell types constitute the contractile myocardium of the atria and ventricles, while cells of the cardiac conduction system and the autonomous nervous system innervate the heart tissues and coordinate rhythmic contractions of the heart chambers.^2^ The two pacemakers, sinoatrial (SA) and atrioventricular (AV) nodes, spontaneously generate electrical impulses driving heart contraction.^3^ Endothelial cells form the inner endocardial lining of the heart lumen,^4^ a subset of which are further specialized to form the heart valves,^5^ while a separate population makes up the coronary blood vessels that supply oxygen for myocardial contraction.^6^ The epicardium provides a protective layer surrounding the heart muscles.^7^ Besides these main sublineages, other cell types, mainly fibroblasts and mesenchyme, provide the structural matrix of the organ and play multiple roles in physiological processes related to cardiac function and regeneration.^8^ The heart also performs other functions such as endocrine and iron homeostasis (reviewed in^9^^,^^10^) which are established though less well described.

The core genetic program and stepwise morphogenesis involved in the development of the heart is largely conserved across metazoans.^11^ Cells making up the heart arise from a pool of common mesodermal progenitors which are specified to the various major lineages. In the zebrafish, these progenitors can be detected by 12 hours postfertilization (hpf) by the expression of *myl7* and *nkx2.5*. Subpopulations of myocardial progenitors could be further distinguished between atrial (expressing *myh6*) and ventricular (expressing *myh7*) myocardium.^12^ Concurrently, endocardial progenitors denoted by the expression of *kdrl* and *cdh5* could be found anterior to the myocardial progenitors.^13^ As the myocardial progenitors migrate to the midline to form a heart tube by 19 hpf, endocardial progenitors proceed to migrate toward the median and line the lumen of the heart tube.^14^ By 22 hpf, a linear, beating heart tube is formed, which is continuously elongated by the addition of cells originating from the second heart field (SHF), a pool of late-differentiating mesodermal progenitors, extending the atrium and ventricle and forming inflow and outflow tracts.^15^^,^^16^ Between 24 and 30 hpf, neural crest cells (NCCs) migrating through the pharyngeal arches contribute to the myocardium of the primitive heart tube, while a second population arriving at 80 hpf contribute to the outflow tract structures.^17^ The NCCs also contribute to the cardiac peripheral nervous system.^18^ Finally, the proepicardial organ forms at 48 hpf close to the atrioventricular junction and can be detected by the expression of *wt1* and *tcf21* markers. This structure gives rise to the epicardial cells which subsequently migrate and envelop the heart.^7^

Although key insights into heart development and function have been derived from the zebrafish model organism,^11^^,^^13^ critical differences exist between the zebrafish and mammalian heart. Unlike mammals, teleosts including the zebrafish possess a heart comprising two chambers. An additional structure unique to teleosts is the bulbus arteriosus (BA), a chamber-like structure similar to the distal outflow of mammalian heart and serves to absorb pressure.^19^ In terms of electrophysiology, the zebrafish heart is fundamentally similar to that of humans, which enables faithful modeling of diseases of the cardiac conduction system.^20^^,^^21^ Yet, differences in terms of ion channels and the types of currents involved in driving cardiac contractions exist.^22^ With the growing use of the zebrafish to model human heart biology, a comprehensive knowledge of both conserved and nonconserved features between the hearts of the two organisms becomes necessary in order to more accurately translate results from the zebrafish to human.

Single cell analyses of mammalian heart have revealed surprising new insights in the discovery of new cell types and their contributions to various forms of heart disease.^23^^,^^24^^,^^25^^,^^26^ The zebrafish offers a glimpse into earlier events of cardiogenesis which could provide valuable insights into the mechanism underlying the development of various cardiac cell types and specialized structures. Several single cell level analyses have been performed in zebrafish which included the heart.^27^^,^^28^^,^^29^^,^^30^ However, these were either focused on selected cell types or have not reached sufficient depth to comprehensively capture and annotate cardiac cell subtypes, particularly rare cell types such as the cardiac pacemaker cells. These cells are embedded within the myocardium and play a central role in generating and propagating electrical impulses for a rhythmic heart contraction. Although previous bulk-level analyses have shed some light into the molecular mechanism of their function,^31^^,^^32^^,^^33^ their scarcity and the lack of defining morphological and molecular features continue to pose a challenge to isolate pure populations of this cell type and study them.

Here, we present a high-resolution atlas of the developing zebrafish whole heart single cell transcriptomics, aiming at sufficient depth to allow discovery and annotation of cardiac cell subtypes. We delineated major cell lineages and sublineages of the zebrafish heart and distinguished a set of gene expression profiles associated with each of these populations. We uncovered a population of cardiomyocytes representing the SA and AV pacemaker cells and defined their unique molecular profile and developmental trajectory. Clustering analyses revealed two novel genes specifically enriched in the primary SA pacemaker, *colec10* and *atp1b3b*, which encode the collectin subfamily member 10^34^ and a subunit of the Na+/K+ ATPase beta chain proteins,^35^ respectively. Loss of function analyses further revealed their role in heart development and rhythm maintenance. Our study established the heterogeneity of zebrafish cardiac cell types which could serve as a valuable resource for future in-depth analyses of cell populations with higher specificity.

## Results

### Isolation and transcriptome profiling of single cells from the embryonic zebrafish heart

**Figure 1:**
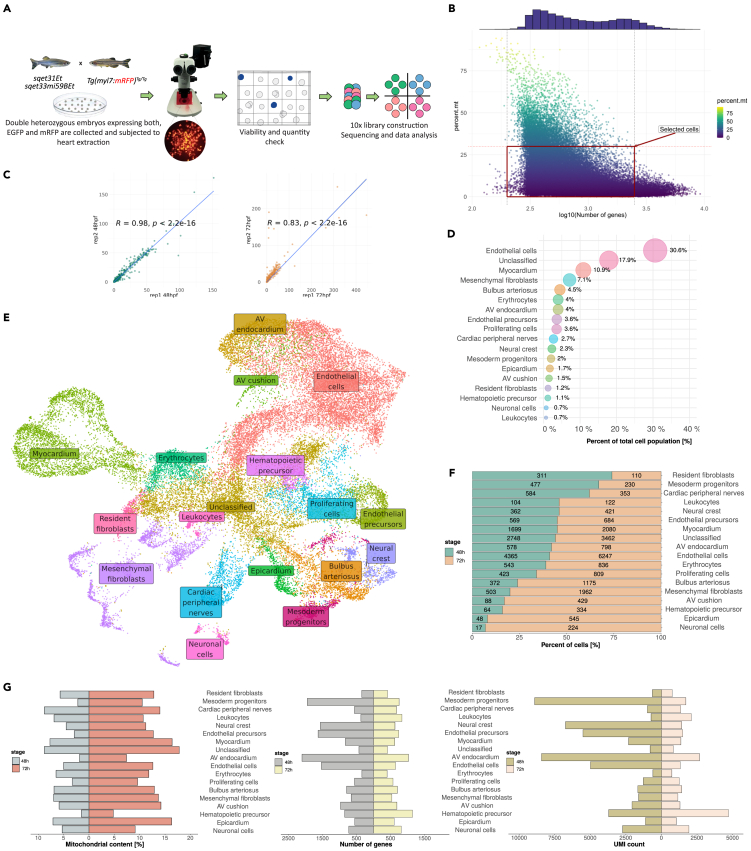
Single-cell RNA sequencing revealed 18 distinct cell subtypes of the embryonic zebrafish heart (A) Schematic overview of experimental design and workflow. (B) Quality parameters of all cells derived from 48 to 72 hpf pseudo-replicates showing the number of expressed genes and mitochondrial gene content. Cells within the red frame were included in the downstream analysis. (C) Pearson correlation between developmental pseudo-replicates at both 48 hpf and 72 hpf. (D) Percent of total cell population contributing to each cluster. (E) Integrated UMAP projection depicting cell clusters that constitute the embryonic zebrafish heart. (F) Visual representation of cluster composition expressed as a percentage indicating the number of cells contributing to each cluster depending on developmental stage. (G) General quality metrics reflecting the percent of mitochondrial genes, the total number of genes, and UMI expressed in each cell cluster at a particular developmental stage.

To generate homogeneous, viable single cell suspension from the zebrafish heart at 48 hpf and 72 hpf, we optimized a cell dissociation protocol incorporating simultaneous trypsin and collagenase treatment. We obtained cell suspension with viability above 90% (Figure S1A). To provide an internal control of specific rare cardiac cell population, we utilized two transgenic lines *sqet33mi59BEt*^32^^,^^36^ and *sqet31Et*, in which cells of the sinoatrial (SA) and atrioventricular (AV) pacemaker regions expressed EGFP.^33^^,^^37^ In order to additionally demarcate the major cell types of the heart, we utilized the *Tg(myl7:mRFP)* transgenic line^38^ to confidently mark cardiomyocytes (CMs), which is the most technically challenging cell type to isolate, and enhance cell clustering. To profile the transcriptome of the zebrafish heart, we collected offspring from two independent crosses of *Tg(myl7:mRFP)* line with either *sqet33mi59BEt* or *sqet31Et* transgenic lines. From each of the double transgenic individual pools, we isolated whole hearts at 48 hpf and 72 hpf and dissociated them into single cells which were subsequently encapsulated according to the 10× Genomics workflow (Figure 1A).

In total, we obtained 52 447 cells from all four biological samples (Figure 1B; Table S1). In each, over 22 000 genes were identified. The median number of genes per cell varied between the developmental stages, which was on average nearly three times higher at 48 hpf as compared to 72 hpf. Similar trend was observed in terms of unique transcript molecules per cell, whereas mitochondrial gene content was double in cells derived from 72 hpf (Figure S1B). At the same time, the Pearson correlation coefficient between raw developmental pseudo-replicates was equal to 0.98 and 0.83 for 48 hpf and 72 hpf data, respectively (Figure 1C). The number of cells after quality control for library size, mitochondrial gene content and possible multiplets ranged from 6728 to 11 015 per sample, resulting in a final number of 34 676 cells for downstream analysis (Table S1). Out of these, 2573 (74% of total *RFP*+ cells) cells co-expressed *RFP* and well established gene signatures for myocardial cells which included *cmlc1* and *myl7*. These form a separate cluster, suggesting cardiomyocytes identity (Figures 1E and S1C). In addition, 136 cells were found to express *EGFP,* out of which the vast majority comes from 48 hpf dataset (90 cells at 48 hpf vs. 46 cells at 72 hpf). Most of the total EGFP+ cells (89) were within the cardiomyocytes cluster, suggesting that our protocol enabled the isolation of single cardiomyocytes, with sufficient sensitivity to capture rare cell populations making up the two pacemaker regions. To facilitate in-depth exploration of our data, we developed an R Shiny application based tool for interactive data visualization, differential expression as well as gene enrichment analysis which is available at https://www.zfcardioscape.iimcb.gov.pl.

### The cellular landscape of the developing zebrafish heart

We distinguished 18 discrete cell populations comprising the zebrafish embryonic heart from both 48 hpf and 72 hpf (Figure 1E). The most abundant clusters comprised endothelial cells (31%), cardiomyocytes (11%) and mesenchymal fibroblasts (7%) (Figures 1D; Table S4). Moreover, the number of cells contributing to each cluster varied between the developmental stages. Clusters originating mostly from 48 hpf included resident fibroblasts, mesoderm progenitors and cardiac peripheral nerves. On the other hand, seven clusters showed a higher proportion of cells derived from 72 hpf. These included neuronal cells, epicardial cells and Bulbus arteriosus (Figures 1F; Table S4). On the other hand, the number of expressed genes and UMIs were greater at 48 hpf as compared to 72 hpf. Among clusters with the highest number of genes and UMIs were Mesoderm progenitors, AV endocardium and Neural crest cells (Figure 1G).

**Figure 2:**
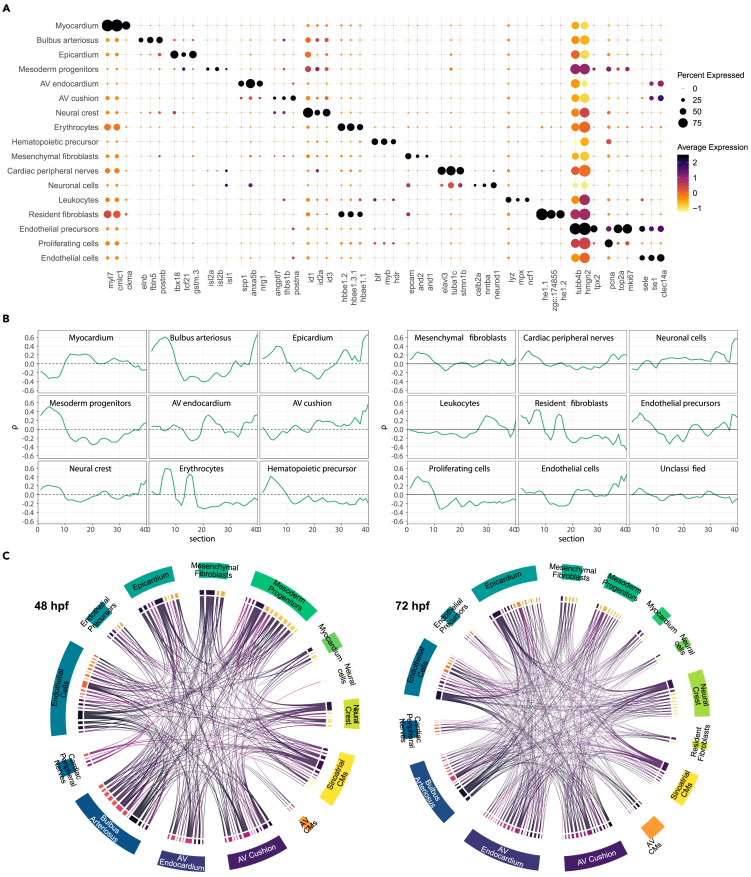
The expression profiles of main cardiac cell clusters correlate with heart structures of known spatial localization (A) Dotplot showing the top three differentially expressed genes for all cell clusters except “Unclassified”. (B) Spatial projection of cardiac single cell expression profiles to zebrafish heart sections derived from Burkhard et al.^31^ at 48 hpf. (C) Interaction map between cell clusters according to ligand-receptor expression. Each line connect individual ligand with its corresponding receptor (source data: Table S7).

Based on the expression of unique marker genes, these clusters could be broadly grouped into the major cell lineages of the developing heart, namely: myocardial, endocardial, epicardial, neural and neural crest, and mesenchymal/fibroblasts. The myocardial lineage formed a distinct cluster of cells represented by the cluster “Myocardium” which could be distinguished by the expression of *myl7, cmlc1, myh6, myh7, tnnt2a* (Figure 2A; Table S5),^39^^,^^40^ as well as the *RFP* transgenic marker (Figure S1C). The endocardial lineage constituted the largest proportion of the cells in our data. These included the clusters “Endothelial cells” and “Endothelial precursors” which express *cdh5*, *tie1* and *fli1*,^41^^,^^42^ as well as the “AV endocardium” and “AV cushion” which contributes to the developing AV valve (Figure 1F; Table S5). In addition to endocardial markers, the latter two express AV canal restricted markers such as *anxa5b* and *nrg1*.^31^^,^^33^ The “AV cushion” differs from the “AV endocardium” cluster by the higher expression of cell adhesion and extracellular matrix proteins (*postna*, *col1a1a/b/2*) (Table S5). The epicardial lineage forms a single cluster “Epicardium” characterized by the expression of *tbx18, gstm.3, wt1b,* and *tcf21*.^28^

Cells of the “Bulbus arteriosus” cluster were distinguished by the expression of *elnb, postnb,* and *fbln5*, reflecting their smooth muscle composition.^19^ Neural and neural crest cells were represented by clusters “Cardiac peripheral nerves”' and “Neuronal cells”' characterized by the expression of *tuba1c* and *elavl3*^43^ and cluster “Neural crest” by the expression of *twist1a/b, id2a, id3*, and *sox9b*.^44^^,^^45^^,^^46^ Mesenchyme and fibroblasts make up a significant population of cardiac cells and could be distinguished by the expression of adhesion molecules such as *cldnh,* and *epcam*.^47^ Two clusters constituting this cell lineage were “Resident fibroblasts” and “Mesenchymal fibroblasts”, the latter of which expressed higher numbers of genes encoding structural proteins and extracellular matrix components such as *col1a1a*, *cdh1,* and those encoding the Claudin family^48^ (Table S5). Interestingly, we also detected a cluster of cells designated as “Mesoderm progenitors” (Figures 1E and 2A) which was enriched in the expression of *isl1, isl2b*, and *ldb1a* which are known to be expressed in cells originating from the second heart field.^16^^,^^49^ Cells of this cluster also expressed a large number of transcription factors and cell cycle regulators (Figure S1D; Table S5). Interestingly, we also captured cells enriched in the expression of hematopoietic markers *blf* and *myb*.^50^^,^^51^ From these, distinct populations enriched in markers of erythrocytes (hemoglobin genes as well as *alas2, hemgn*^52^) and leukocytes comprising of neutrophils (*mpx*,^53^ *ncf1*^54^), macrophages (*mpeg*^55^), and platelets (*mpl, itga2b*^56^) could be distinguished (Figures 2A; Table S5).

Different cell clusters exhibited variable proliferation activity based on the expression of genes driving the G2M and S-phase of the cell cycle. The most proliferative cluster being “Endothelial precursors”, the cells of which expressed a large number of G2M and S-phase related genes (Figure S1D). The clusters “Bulbus arteriosus”, “Leukocytes”, “Mesoderm progenitors'', “Erythrocytes”, and “Resident fibroblasts” were found to be more proliferative at 48 hpf compared to 72 hpf (Figure S1D), which may reflect their differentiation process. Accordingly, clusters expressing known cell-type-specific markers were generally low in proliferation activity (<50%). These include “Myocardium”, “AV cushion” and “AV endocardium”, “Endothelial cells”, and “Neuronal cells” (Figure S1D).

In order to provide spatial context to the cell clusters, we took advantage of the available spatial information from the serial cross-section transcriptome dataset of the zebrafish heart.^31^ Correlating the expression profiles of our main scRNA-seq clusters with that of each serial section, the analysis revealed that cell types associated with structures known to be spatially localized exhibited the highest correlation with sections representing their corresponding localization (Figure 2B). Furthermore, spatial correlation between each main cluster and their corresponding fine-grained clusters (see supplementary method) revealed overall high levels of both gene and spatial cluster correlation, except for the “Unclassified” cluster which was heterogeneous in both spatial and gene expression values, and the “Mesenchymal fibroblasts” cluster which was homogeneous in terms of gene expression values but spatially heterogeneous (Figure S2; Table S6). This suggested that these clusters might be controlling subparts of the main clusters which have a differentiated role but are otherwise attached to the developmental structure associated with the respective main cluster. Taken together, the spatial correlation analyses of the scRNA-seq clusters agree with the spatially restricted expression profile, thereby providing additional support for our cluster annotation.

To identify potential cellular crosstalk between cell types in the developing heart, we constructed an interactome map between the cardiac cell clusters based on the expression of ligands and their receptors.^57^ The analysis revealed several known cellular interactions including those involved in the development of AV cushion.^58^ Genes encoding Bmp4/5/6 were expressed in the “AV cardiomyocytes” cluster, while those encoding its signaling receptors Bmpr2a, Acvr1ba, and Sdc2 were expressed in the “AV cushion” cluster (Figure 2C; Table S7). The analyses also suggested potential interactions which were not previously established. For instance, the cluster “Cardiac peripheral nerves” interacted with “Endothelial cells” and “Epicardium” clusters through Robo1/2 and its receptor Slit1a/2/3 expressed in the corresponding clusters (Figure 2C; Table S7). The Robo/Slit signaling was implicated in axon guidance, as well as in endocardial progenitors migration to the midline.^59^ The “Cardiac peripheral nerves” cluster also interacted with “SA cardiomyocytes” subcluster through several known ligand-receptor pairs including Plxna3 - Sema3aa, Fgfr2/3/4 - Ncam1a, and Sdc4 - Mdkb (Figure 2C; Table S7), which have been implicated in neural patterning.^60^^,^^61^ Taken together, the analysis revealed potential interactions between cell or tissue types and their underlying molecular players for further in-depth analyses.

### Diverse myocardial cell populations constitute chamber myocardium and the cardiac conduction system

**Figure 3:**
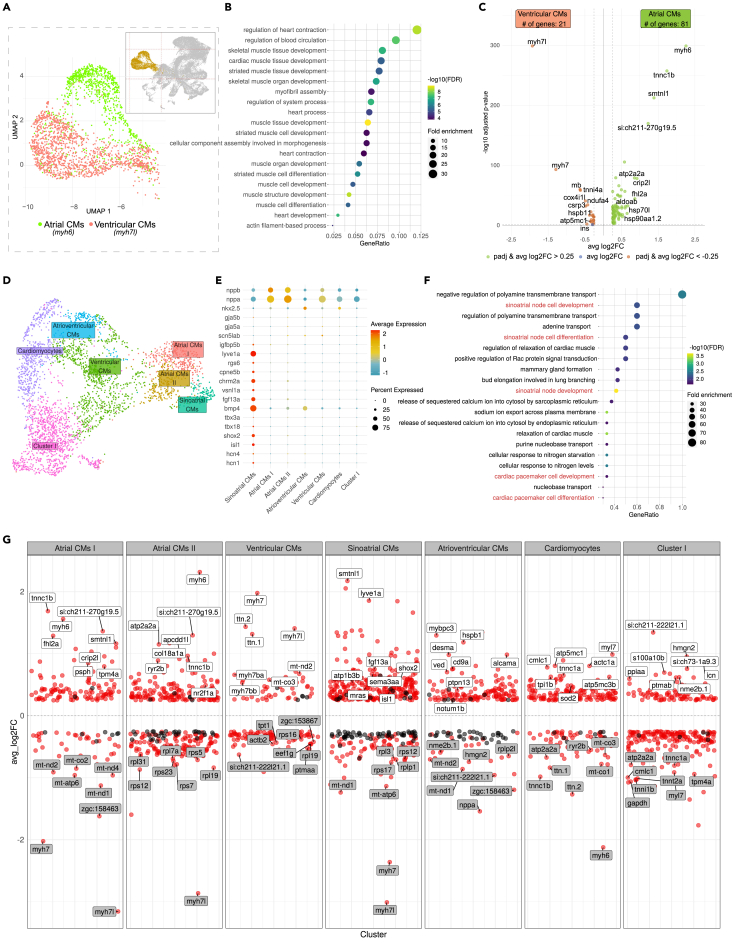
Analysis of the “Myocardium” cluster revealed the diversity of myocardial cells and pinpointed sinoatrial cardiomyocytes within the atrial myocardium (A) Two distinct identities of chamber myocardium could be delineated by molecular markers: Atrial CMs by expression of *myh6* and Ventricular CMs by *myh7l* expression. (B) Gene Ontology enrichment analysis of all genes enriched within the “Myocardium” cluster. (C) Volcano plot depicting differentially expressed genes between atrial and ventricular CMs fractions. (D) UMAP projection of re-clustered myocardial cells reflecting the heterogeneity of atrial and ventricular myocardium. (E) Dotplot showing the expression (SCT normalized counts) of well-established gene signatures associated with working myocardium and sinoatrial pacemaker within “Myocardium” clusters. (F) Gene Ontology enrichment analysis of genes enriched within the “Sinoatrial CMs” subcluster. (G) Differentially expressed genes in each myocardial subclusters. Top 8 genes in terms of significance were labeled. Genes with adjusted *p*-value <0.05 are depicted as red dots while not significant genes (adjusted *p*-value >0.05) are shown in black. Source data: Tables S8, S9, and S10.

The myocardium is the main tissue type of the heart which is responsible for the organ’s main contractile function. Myocardial cells are characterized by their contractility and high metabolism.^62^ In agreement with this, Gene Ontology terms related to muscle function and structure were enriched among genes in this cluster (Figures 3A and 3B; Table S8). Chamber cardiomyocytes could be distinguished by the expression of well-established marker genes.^63^^,^^64^ Within the cluster “Myocardium”, a clear distinction between atrial and ventricular cardiomyocytes was evident by the complementary expression of atrial (*myh6*) and ventricular (*myh7* and *myh7l*) cardiomyocyte markers (Figure 3A). Differential expression analysis between both fractions confirmed the presence of a set of unique genes in each heart chambers. The top differentially expressed signatures comprised genes encoding for various structural proteins involved in active force generation, including *myh6, tnnc1b* and *smtln1* (atrial), and *myh7l*, *myh7*, and *tnni4a* (ventricular) (Figure 3C).

In order to delineate subpopulations of myocardial cells at higher resolution, we further re-clustered cells within the “Myocardium” cluster (a total of 3315 cells) (Figure 3D). The analysis revealed that at least 43% of myocardial cell populations consisted of those making up the working myocardium of the two cardiac chambers, namely, atrial (“Atrial CMs I” and “Atrial CMs II”) and ventricular (“Ventricular CMs”). The mutually exclusive expression of *myh6* and *myh7/myh7l*, together with other unique markers, differentiate these two subtypes of chamber myocardium (Figure 3G; Table S9). Two distinct subclusters of atrial cardiomyocytes were different in terms of higher mitochondrial content in “Atrial CMs II” compared to “Atrial CMs I” (Figure S1E). In addition, “Atrial CMs II” was enriched for the expression of *myoz2b*; Table S9), the mammalian ortholog of which was previously reported to mark a distinct population of cardiomyocytes with yet unknown function.^65^ An additional cluster designated as “Cardiomyocytes” expressed *myl7* which identified these cells as cardiomyocytes. However, they are not enriched in any chamber-specific markers.

Besides the working myocardium, the cardiac conduction system constitutes an important structure of myocardial origin. Among cells with high expression of atrial-specific gene (*myh6*), we identified a group of 127 cells that uniquely express *lyve1a*^31^*, vsnl1a*^66^, and *chrm2a*^67^ that have been reported to be upregulated in the heart SA region. These cells also express other genes implicated in the function of the SA pacemaker, including *isl1, shox2, hcn4, bmp4, fgf13a, tbx18*,^31^^,^^32^^,^^68^ while maintaining low expression of working myocytes markers, e.g., *nppa/b, nkx2.5, gja5a/b* (Figure 3E). Gene Ontology enrichment analysis revealed numerous GO terms related to the sinoatrial node and cardiac pacemaker cell development (Figure 3F; Table S10). We therefore define this cluster as “Sinoatrial CMs” that could represent a group of specialized cardiomyocytes of the primary pacemaker site of the cardiac conduction system. Similarly, the cluster “Atrioventricular CMs” is distinguished by the expression of *bmp4*, and *tbx2b* (Figures 3E and 3G; Table S9), which were previously associated with the atrioventricular canal.^33^ These two pacemaker subclusters were enriched for partially overlapping sets of genes encoding transcription factors, ion channels, as well as cell adhesion molecules (Figure S3; Table S11), in line with our previous results from bulk transcriptome analysis.^32^^,^^33^

To determine the relevance of our single cell transcriptome data as a potential resource for studying human heart diseases, we explored whether any clinically relevant SNPs have been reported in the human orthologues of genes enriched in various myocardial clusters. Utilizing the ClinVar database,^69^ we found a total of 52 genes in the collective myocardial cluster to be associated with heart conditions related to conduction, arrhythmia, AV block, and long QT syndrome (Table S12). Among these, 23 genes were enriched in the “sinoatrial CMs” and 18 in the “atrioventricular CMs” clusters.

### Functional analysis of cell-type-specific markers reveal new potential players in heart rhythm maintenance

**Figure 4:**
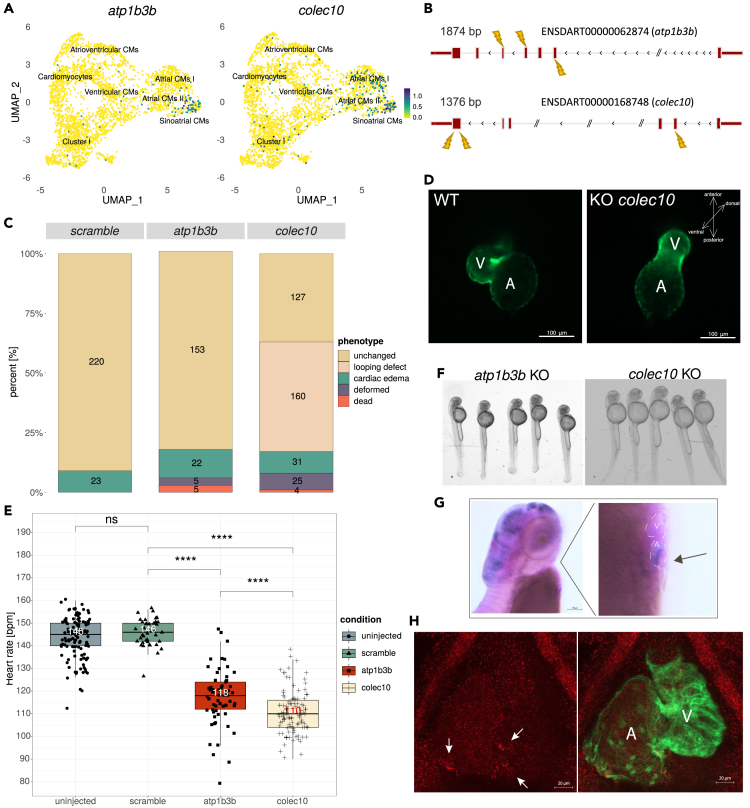
Functional analysis identified atp1b3b and colec10 as new players in heartbeat maintenance (A) Expression of *atp1b3b* and *colec10* within the myocardial subclusters. The highest expression for both genes was observed within Sinoatrial CMs cluster. (B) Scheme illustrating gene loci targeted by CRISPR/Cas9 strategy. The combination of three sgRNAs were utilized for *atp1b3b* and *colec10* knockout generation. Control embryos were injected with a scrambled sgRNA. (C) Barplot summarizing observed phenotypes from all replicates for each gene knockout. Numbers within each section indicate the number of embryos associated with a particular phenotype. (D) Fluorescent images showing heart looping defect in *colec10* knockouts as compared to uninjected embryos (WT represents embryos on *Tg(myl7:EGFP-Hsa.HRAS)*^*s883*^ background at 48 hpf). (E) Heartbeat rate comparison between appropriate knockouts, uninjected wild-type control, and scrambled-injected embryos (significance values according to Wilcoxon rank-sum test). (F) Representative image showing morphology of *atp1b3b* and *colec10* knockout embryos. (G) Whole mount *in situ* hybridization of *atp1b3b*. (H) Hybridization chain reaction *in situ* for *atp1b3b* performed on 3 dpf larvae. Note the restricted heart expression within the sinoatrial region (G, H). Detailed single confocal plane images provided in the Figure S4A.

To identify novel molecular players involved in the development and function of the primary pacemaker, we searched for genes which are specifically enriched in the “Sinoatrial CMs” cell cluster and identified two genes, *atp1b3b* and *colec10* (Figure 4A; Table S9). Whole mount *in situ* hybridization and hybridization chain reaction revealed that the expression of *atp1b3b* in the heart were found to be particularly restricted to the sinoatrial region (Figures 4G, 4H, and S4A), which suggested this gene as a novel marker for pacemaker cells in the zebrafish. On the other hand, *colec10* expression was undetectable by these two methods (Figure S4B). To establish the function of these two novel candidate genes, we performed loss of function analysis by the CRISPR/Cas9 based F0 knockout.^70^^,^^71^ To target *atp1b3b,* we designed sgRNAs against the 2nd, 4th and 5th exons (Figures 4B and S5). By 48 hpf, loss of function of *atp1b3b* did not appear to affect heart development nor overall morphology (Figures 4C and 4F). Targeting of *colec10* by three sgRNAs directed against the 2nd and 6th exons (Figures 4B and S5; Table S2) caused a high proportion of embryos with hearts that failed to loop by 48 hpf (46%, *n* = 160/347, Figures 4C and 4D). These embryos appeared morphologically normal otherwise, with only a very small number of embryos exhibiting gross morphological abnormality (Figures 4C and 4F).

The specific expression of *atp1b3b* and *colec10* in the “Sinoatrial CMs” cluster representing the primary pacemaker cell population led us to question whether they are involved in regulating heart rhythm. We systematically calculated the heartbeat rate in CRISPR/Cas9-injected embryos (Figures 4E and S6; Table S13). While the scrambled sgRNA did not induce any observable phenotype nor reduction in heart rate, heartbeat rate was significantly lower in both *atp1b3b* and *colec10* knockout compared to uninjected and scrambled-injected siblings (Figures 4E, Videos S1 and S2). By 48 hpf, knockout of *atp1b3b* resulted in a median of 118 bpm which was 28 heart beats per minute (bpm) less compared to scrambled-injected siblings (*n* = 61, *p*-value = 9.4e-16). However, at 72 hpf, the reduction in heart rate becomes insignificant (*n* = 12, *p*-value = 0.0643; Figure S6; Table S13). On the other hand, in *colec10* knockout, the larvae had a heart rate of 110 bpm by 48 hpf, which is 36 bpm less compared to scrambled-injected siblings (*n* = 109, *p*-value = 2.2e-16) (Figure 4E). The reduced heart rate is retained at 72 hpf (*n* = 12, *p*-value = 0.0004; Figure S6; Table S13). The genetic mozaicism of these F0 individuals limited our ability to ascertain the true function of the two candidate genes. Hence, more detailed analysis of stable mutants is necessary and underway. Collectively, these observations suggest that the two newly identified sinoatrial pacemaker genes play a role in heart development and regulation of heart rhythm.

Video S1. Comparison of heartbeat rate in atp1b3b CRISPR knock-out and control, related to Figure 4 and S6

Video S2. Comparison of heartbeat rate in colec10 CRISPR knock-out and control, related to Figure 4 and S6

### The cardiac endothelial cell subpopulations possess distinct molecular profiles

**Figure 5:**
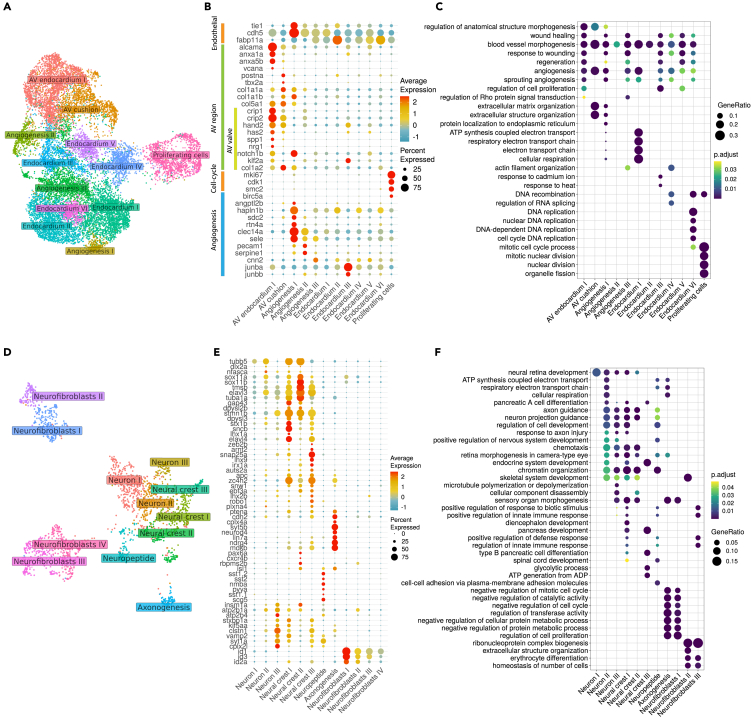
Molecular profiles of diverse endothelial and neural cell subclusters UMAP projection of endothelial (A) and neural (D) subclusters. Dotplot showing the expression of specific marker gene signatures associated with various endothelial (B) or neural (E) cell subclusters. Average expression values represent SCT normalized counts, percent expressed values represent the proportion of cells within a cluster that expresses a particular gene. Gene Ontology enrichment analysis of each endothelial (C) and neural (F) subclusters depicting the enriched functional terms (GO level 5) in each cluster. Adjusted *p*-values (Holm–Bonferroni) are indicated in color, gene ratio represents the gene count mapped into each GO category. Source data: Tables S14, S15, S16, and S17.

Endothelial cells perform critical function in heart development and physiology and are known to contribute to different heart tissues including cardiac valve, coronary vessels, and trabeculae.^72^ In order to characterize the diversity of cardiac endothelial cells in the developing heart, we re-clustered cells of the “Endothelial cells”, “Endothelial precursors”, “AV endocardium”, and “AV cushion” clusters. Our analyses revealed that the cardiac endothelial cells consisted of molecularly distinct populations (Figure 5A). Clusters “AV endocardium” and “AV cushion” were distinguished by the expression of several genes characteristic of the endocardium of the AV region, which included *hand2, has2,* and *crip2*^33^^,^^58^^,^^73^ (Figure 5B). Cluster “AV endocardium” genes included *anxa1a* and *anxa5b*, both of which are known to be expressed in AV canal region,^31^ as well as *notch1b* and *klf2a* which are early markers of the AV valve^58^ (Figure 5B). Cluster “AV cushion” was enriched in genes coding for extracellular matrix constituents including many collagen family (*col1a1b, col1a2*, *col5a1/2a,* and *thbs1a/b*), as well as *postna* which are known to be expressed in the AV cushion.^33^ This cluster also had the highest expression of *col1a2* which is implicated in human cardiac valve disease^74^ (Figure 5B; Table S14).

Noticeably, several of the endothelial subclusters were significantly enriched in functional terms related to blood vessel morphogenesis and angiogenesis (Figure 5C; Table S15). Cluster “Proliferating cells” was significantly enriched in the expression of genes encoding cell cycle regulators (Figure 5C; Table S5). Cells of this cluster uniquely express *birc5a* which is known for its role in vasculogenesis and angiogenesis.^75^ Cells of “Angiogenesis I” cluster expressed the most number of genes implicated in sprouting angiogenesis, including *hapln1b*^76^*, sdc2*^77^, and *rtn4a*^78^ (Figure 5B). Cluster “Angiogenesis II” shared a partially overlapping set of angiogenesis genes with “Angiogenesis I″, such as *clec14a*,^79^ as well as *sele*,^80^ *pecam1*^81^ and *serpine1*.^82^ Apart from *sele* and *serpine1*, cluster “Angiogenesis III” also expressed several unique genes including *cnn2* which is implicated in blood vessel endothelial cell migration^83^ (Figure 5B). The rest of the endothelial cell clusters were not enriched in any specific functional category other than that which suggest their endothelial identity and hence are likely to be endocardial lining of the cardiac chambers. Collectively, the diversity of the cardiac endothelial cell population reflects their contribution to various structures of the heart and suggests their functional diversity.

### Neural crest and the intracardiac nervous system

Besides the main cardiac cell lineages, we also identified other cell populations whose expression profiles we provide as a resource (Table S5). One important but poorly characterized cell populations are those making up the cardiac peripheral nervous system, which ensures the regulation of heart contraction speed according to physiological demands.^84^ To evaluate the diversity of the neural and neural crest cell populations in the zebrafish heart, we re-analyzed the main clusters “Cardiac peripheral nerves”, “Neural crest” and “Neuronal cells”. We distinguished subpopulations of cells which could be broadly subdivided into neuronal and non-neuronal cells (Figure 5D). Clusters “Neurons I-III” and “Neural crest I-III” were enriched in the expression of neuro differentiation markers including *elavl3, elavl4, stmn1b, tuba1a,* and *tubb5*^43^^,^^85^^,^^86^^,^^87^ (Figures 5E and 5F; Table S16). They were also enriched in the expression of *sncb* which is involved in dopaminergic neuron differentiation and development of motor functions.^88^ Clusters “Neural crest I-III” were additionally enriched in the expression of several genes implicated in neural crest development, including *mdkb*, *pbx4*, *tfap2a*, and *snw1*.^89^^,^^90^^,^^91^^,^^92^ Cluster “Neuropeptide” was enriched in the expression of genes encoding neuropeptide signaling molecules (*npb*^93^*, scg5*^94^*, nmba,* and *pyya*^95^), as well as factors promoting dendrite morphogenesis and cell migration (*sst1.1/1.2/2*,^96^ and *tmsb*^97^) (Figures 5E; Table S16). The “Axonogenesis” cluster expressed genes encoding proteins known to be associated with axons or denrites including *dscaml1, tnc, draxin,* and *nptnb*.^98^^,^^99^^,^^100^

Compared to other neural subclusters, clusters “Neurofibroblasts I-IV” were depleted in neural and neural crest markers expression while enriched in the expression of *id1* and *id3* known for their role as inhibitors of neurogenesis^101^ (Figures 5E; Table S16). Clusters “Neurofibroblasts I” and “Neurofibroblasts II” expressed a large number of genes implicated in cell cycle such as *jun/ba/bb/d* and *cdkn1bb*. They also uniquely expressed *egr1* which is implicated in neural stem cell maintenance,^102^ suggesting the characteristics of neuron stem-like cells. Hence, their expression profiles suggest their identity as neural fibroblast cells. Cluster “Neurofibroblasts III” was enriched in genes coding for the most number of extracellular matrix components including collagens and keratins, which suggests structural function Table S17. Together with “Neurofibroblasts IV” cluster, it was also enriched for *postnb* encoding the zebrafish ortholog of Periostin which is known to be expressed in endoneurial cardiac fibroblasts contributing to sympathetic nerve fasciculation in the mammalian heart.^103^ Taken together, the expression resource for these clusters, as well as others, could potentially reveal novel cardiac cell types which are poorly explored and open new pathways of investigations.

## Discussion

We present a high-resolution atlas of the developing zebrafish heart between 48 hpf and 72 hpf obtained through single cell transcriptomics. The developmental stages chosen constitute a critical phase of heart development, where specialized cardiac structures are formed, including the atrioventricular canal, heart looping, as well as the cardiac valves and conduction system.^58^^,^^104^ In addition, this developmental period also covered the time when external cell types are incorporated into the heart, forming structures such as the cardiac peripheral nerves and the proepicardium.^7^^,^^17^

CMs are widely recognized as one of the most challenging cell types to isolate (discussed in Paik et al^105^). While we cannot rule out the potential bias in terms of their survivability, our protocol enabled the capture of sufficient numbers of this cell type. This not only allowed for the determination of cluster identity but also provided the confidence to distinguish cell subpopulations based on the significant expression of known marker genes. Furthermore, despite occupying the largest volume of the heart, numerous studies have established that CMs rank as the 2nd or 3rd most abundant cell type in the mammalian heart in terms of number after cardiac endothelial cells, constituting approximately 30% and 50%, respectively (discussed in Zhou and Pu^106^). Our dataset generally aligns with this consensus, reflecting the relative abundance of these cell populations (Table S4).

To retain high-quality cells for downstream analysis, we applied multiplets removal and stringent quality check thresholds. Despite our best effort to refer to available public resources and literature, we were unable to establish the identity of cells contributing to the "Unclassified" cluster which constitutes the second-largest cell population in our dataset. Nevertheless, these cells successfully passed the applied QC thresholds, therefore, we decided to retain them since we believe the data removal should be made with extra care. We suspect that these cells may still present a meaningful value when analyzed in smaller clusters at higher clustering granularity.

Our study delineated major cardiac cell lineages which were supported by known molecular markers. In addition, spatial correlation analysis points to main clusters which possess specific anterior-posterior localization within the heart tube, which provided further support of their identity. Further correlation analysis between the main and subcluster components (fine-grained clusters at higher resolution, see supplementary methods) revealed the homogeneous composition of several main clusters, while pointing out the heterogeneous composition of other clusters, such as the mesenchymal fibroblasts, on which scarce literature information is available and annotation is therefore less precise. To date, there is only one published spatial transcriptomic data on the embryonic zebrafish heart.^31^ Although the section-based method limits the cellular resolution, it provided a reliable positional reference for major cardiac structures. Future spatial analysis at higher resolution is expected to enable the annotation for each cell type with improved accuracy.

Our analyses uncovered subpopulations within the major cardiac cell lineages, including previously uncharacterized subtypes of cells, along with their molecular profiles. Previous single cell analyses on the developing epicardium in zebrafish has uncovered distinct subpopulations, each possessing specific genetic signatures and function.^28^ Similarly, transcriptome profiling of mammalian cardiomyocytes at the single cell level have revealed unprecedented molecular diversity which seems to suggest further functional specializations and/or distinct energetic profiles within this cell type.^25^^,^^65^^,^^107^ A specific population of cardiomyocytes that express Myoz2, a member of the sarcomeric protein family that is implicated in hypertrophic response,^108^ was recently described in mouse and humans.^26^^,^^65^ We observed that *myoz2b,* a zebrafish ortholog of this gene was expressed in the zebrafish myocardium. *myoz2b* expression were detected in subsets of cells across almost all myocardial clusters. Determining the features and function of this particular cardiomyocyte population would provide key insights into how it contributes to the overall functioning of the heart.

Single cell analysis allowed us the opportunity to identify and study molecular components implicated in the development and function of rare cell populations, including those constituting the primary pacemaker, which high specificity. We functionally characterized two genes, *atp1b3b* and *colec10*, which were specifically enriched in the sinoatrial cardiomyocytes representing the primary pacemaker region. Our loss-of-function analyses revealed the potential role of both genes in heart development and maintaining heart rhythm. The *atp1b3b* gene encodes the ꞵ3 subunit of the Na+/K+ ATPase family responsible for osmoregulation and electrical excitability of nerve and muscle. In the heart, the ɑ1 and ɑ2 isoforms, as well as the ꞵ1 are predominantly expressed.^109^ However, some studies have also reported the expression of ꞵ3 isoform in the ventricular myocytes.^110^ Evidence have suggested that a functional distinction exist between the isoforms, although the exact mechanism is still poorly understood. Interestingly, our single cell transcriptome analysis revealed that *atp1b3b* had the most restricted expression in the sinoatrial CMs compared to other genes encoding the ɑ and ꞵ subunits. This may also underlie the specific heart rhythm phenotype observed upon their loss of function. Future analysis will further distinguish the function and mechanism of the different members of the Na+/K+ ATPases which has potential implications in developing therapies for heart failure. The gene *collectin sub-family member 10 (colec10)* encodes a member of the C-type lectin family,^34^ mutations of which have been associated with the rare 3MC syndrome 3 in humans.^111^ The gene product of *colec10* was implicated in cellular movement and migration *in vitro,*^111^ while scarce information is available on the function of this gene *in vivo*. Our observation of its specific expression in the sinoatrial CMs, together with the defects observed upon its loss of function, therefore suggest that this gene may be a new player in regulating heart development and conduction.

Our single cell transcriptome analyses also captured diverse populations of cardiac endothelial cells and at least two extracardiac cell lineages - the cardiac neural crest and fibroblasts. An interesting observation is that several endothelial cell clusters were found to be enriched for genes associated with blood vessel formation and angiogenesis. It was previously reported that the zebrafish coronary vasculature originates from the endocardial population and develops only from 1 to 2 months post-hatching.^112^ Moreover, endocardial cells are known to share the expression of vascular markers.^113^ Therefore it would seem unlikely that the enrichment of these functional categories reflects an actual process of vasculogenesis. Nevertheless, the distinct molecular profiles of the cardiac endothelial cell populations suggest the presence of functional diversity at the embryonic stage of heart development. Annotation of these cell types, particularly the latter, are still challenging as there are currently very few molecular markers that can be used to define them.^8^ Previous studies in the zebrafish have focused on cardiac fibroblasts within the context of regeneration and hence capture only the activated form.^114^^,^^115^ We nevertheless provide their expression profiles as a resource which is envisaged to open the pathway for further studies of various aspects of cardiac biology in the zebrafish model organism. The upcoming challenge will be to ascribe function to each cell type and determine the extent to which these diversity of cell types are conserved in comparison to the human heart in terms of their molecular properties and function.

### Limitations of the study

• In the CRISPR F0 knockout analysis of *atp1b3b* and *colec10*, the genetic mosaicism limited our ability to ascertain the true mechanism of the two candidate genes. Hence, more detailed analysis of stable mutants is necessary and underway.
• The lack of true spatial transcriptomics reference of the zebrafish heart limited the cellular resolution of our spatial correlation analysis.
• Precise annotation of cell types based on the limited number of characterized marker genes did not allow us to ascertain the cell identities of several cell clusters. We nevertheless provide their expression profiles as a resource in the hope of opening new pathways for further in-depth analyses.
• Considering the sparse nature of single cell expression data, it is reasonable to expect that not all the individual cells within the particular cluster would express all of the identifying markers for the cluster (discussed extensively in the following examples^116^^,^^117^^,^^118^: While this technical feature of scRNA-seq is inevitable, algorithms including Seurat^119^ which we and many other studies have employed, address this sparsity by implementing dimension reduction techniques that borrow strength across genes to identify cell-cell similarities.^120^

## STAR★Methods

### Key resources table

**Table:** 

REAGENT or RESOURCE	SOURCE	IDENTIFIER
**Antibodies**		

Anti-Digoxigenin-AP, Fab fragments	Roche	11093274910; RRID:AB_2313640

**Chemicals, peptides, and recombinant proteins**		

PTU	Sigma-Aldrich	P7629
Tricaine	Sigma-Aldrich	A5040
Leibovitz’s L-15 Medium	Gibco	21083–027
Fetal bovine serum	Sigma-Aldrich	F2442-500ML
Collagenase	Sigma-Aldrich	C0130
Trypsin-EDTA	Corning	25-051-CI
Alt-R™ S.p. Cas9 Nuclease V3	IDT	1081059
proteinase K	Roche	3115836001

**Critical commercial assays**		

Chromium Next GEM Single Cell 3′ Reagent Kits v.3.1	10× Genomics	1000128
10× Chromium Controller Chip G	10× Genomics	1000127
TapeStation	Agilent	G2992AA
High-Sensitivity DNA Kit	Agilent	5067–4626
Qubit dsDNA High-Sensitivity Assay Kit	Invitrogen	Q32851
NextSeq 500/550 High Output Kit v2.5 (150 cycles)	Illumina	20024907
KAPA Library Quantification Kit Illumina® Platforms	Kapa Biosystems/Roche	KR0405
HCR™ RNA-FISH v3.0	Molecular Instruments	N/A

**Deposited data**		

Raw data	This paper	GEO: GSE234216
Transcriptome data visualization	This paper	https://www.zfcardioscape.iimcb.gov.pl

**Experimental models: Organisms/strains**		

Tg(myl7:EGFP)	Huang et al., 2003^121^	ZDB-ALT-050809-20
Tg(myl7:EGFP-Hsa.HRAS)s883	D’Amico et al., 2007^122^	ZDB-ALT-070309-1
Tg(myl7:mRFP)	Rohr et al., 2008^38^	ZDB-ALT-080917-1
sqet31Et	Poon et al., 2010^37^	ZDB-ALT-070702-10
sqet33mi59BEt	Poon et al., 2016^36^	ZDB-ALT-110630-4

**Oligonucleotides**		

See Tables S2 and S3 for oligonucleotides sequences used in this study	N/A	N/A

**Software and algorithms**		

10× Genomics Cellranger pipeline version 3.1.0	10× Genomics	https://support.10xgenomics.com/single-cell-gene-expression/software/overview/welcome
Seurat package v4.0.1	Stuart et al., 2019; Hafemeister et al., 2019	https://satijalab.org/seurat/index.html
DoubletFinder	McGinnis et al., 2019	https://github.com/chris-mcginnis-ucsf/DoubletFinder
pyHeart4fish	Vedder et al., 2023	https://github.com/ToReinberger/pyHeart4Fish
bcl2fastq v2.19.0.316	Illumina	N/A
glmGamPoi	Hafemeister et al., 2019	https://bioconductor.org/packages/release/bioc/html/glmGamPoi.html
ClusterProfiler v. 3.17.3	Yu et al. 2012	https://bioconductor.org/packages/release/bioc/html/clusterProfiler.html
DanioTalk	Chodkowski et al., 2023	https://github.com/DanioTalk
chopchop	Labun et al., 2019	https://chopchop.cbu.uib.no/
ggplot2	Wickham et al., 2009	https://ggplot2.tidyverse.org/

**Other**		

Chromium Controller	10× Genomics	https://www.10xgenomics.com/instruments/chromium-controller
Light cycler 480	Roche	05015278001
Countess 3 automated cell counter	Invitrogen	AMQAX2000
Zeiss LSM 800 confocal microscope	Zeiss	N/A
SZX16 fluorescent stereomicroscope	Olympus	SXZ16-ILLB
acA1440-220 μm USB 3.0 camera	Basler	107652
PicoPump	World Precision Instruments	PV820
2100 Bioanalyzer	Agilent	G2938A
Qubit Fluorometer	Invitrogen	Q32866

### Resource availability

#### Lead contact

Further information and requests for resources and reagents should be directed to and will be fulfilled by the lead contact, Cecilia Winata (cwinata@iimcb.gov.pl).

#### Materials availability

This study did not generate new unique reagents.

#### Data and code availability

• Single-cell RNA-seq data have been deposited at NCBI GEO and are publicly available as of the date of publication with accession number GEO: GSE234216.
• The original code generated in this work for the R shiny application has been deposited at Github and is publicly available as of the date of publication: https://github.com/kanahia/ScExploreR.
• Any additional information required to reanalyze the data reported in this paper is available from the lead contact upon request.

### Experimental model and study participant details

#### Animal husbandry

Zebrafish transgenic lines *sqet31Et*,^33^^,^^36^^,^^36^ *sqet33mi59BEt*,^32^^,^^36^^,^^37^ *Tg(myl7:mRFP)*,^38^ *Tg(myl7:EGFP)*^121^ or *Tg(myl7:EGFP-Hsa.HRAS)*^*s883*^,^122^ were maintained in the zebrafish facility of the International Institute of Molecular and Cell Biology in Warsaw (license no. PL14656251) in line with standard procedures and ethical guidelines. Embryos were raised in egg water at 28°C, screened for fluorescence signals in the heart and staged at 48 hpf and 72 hpf based on established morphological criteria.^123^ All zebrafish embryos utilized for microscopy were supplemented with 0.003% PTU (Sigma-Aldrich, P7629) in E3 medium (5 mM NaCl, 0.17 mM KCL, 0.33 mM CaCl, 0.33 mM MgSO4, pH 7.4) shortly before 24 hpf to inhibit pigmentation and ease heart rate observations.^124^ The zebrafish studies were conducted prior to sexual distinction, hence sex was not determined or considered. While sex differences could potentially influence these findings, especially in older or adult stages, further research could explore how sex-specific factors affect later developmental stages.

### Method details

#### Heart extraction and single cell dissociation

Whole hearts were extracted from double transgenic individuals [*sqet31Et* x *Tg(myl7:mRFP)* and *sqet33mi59BEt* x *Tg(myl7:mRFP)*]. The term “pseudo-replicates” was used to denote samples of two different genotypic backgrounds (*sqet31Et* and *sqet33mi59BEt*) at the same developmental stage. Embryos were anesthetized with Tricaine (0.16 mg/mL in egg water) and large-scale extraction was performed according to a previously published protocol, with minor adjustments.^125^ For each experiment, approximately 1000–1500 larvae were processed for heart isolation. Briefly, dechorionated embryos are resuspended in EDM solution (27 mL of Leibovitz’s L-15 Medium and 3 mL of 10% fetal bovine serum) and aspirated 6–12 times using a 200 μL pipette tips which tips had been cut. The dissociated embryo solution was then filtered through a 70 μm nylon cell strainer (VWR 732–2758) to remove large debris, followed by a second filtration through a 40 μm nylon cell strainer (VWR 732–2757). Retained hearts and other tissue debris were then washed out with EDM into a glass Petri dish. Hearts were manually separated from remaining tissue under a fluorescent stereomicroscope (Olympus) and collected into 0.5 mL of EDM. Typically, 400 to 600 hearts were obtained in each experiment. Single cell heart suspension was obtained according to Bresciani et al.^126^ Briefly, isolated hearts were resuspended in a dissociation mix containing 0.25% Trypsin-EDTA supplemented with 100 mg/mL collagenase and incubated in a thermomixer (Eppendorf) at 30°C for 30 min with gentle pipetting every 5 min using 1 mL low-binding pipette tips (Axygen). The dissociation reaction was stopped by adding ice-cold EDM medium and subjected to two rounds of centrifugation for 5 min at 1000 rpm (Eppendorf 5424 R). The resulting pellet was washed with 0.04% BSA in PBS. To remove the remaining debris, the cell suspension was filtered through a 35 μm FACS tube cell strainer (Falcon, Catalog No. 352235). Ultimately, cells were resuspended by gentle pipetting, loaded on the cell counting chamber, visually inspected under the microscope (Zeiss Apotome), and quantified by Countess automated cell counter (Invitrogen) (Figures 1A, S1A, and S1F).

#### Library prep and sequencing

After viability and quantity check, dissociated cells derived from embryonic zebrafish hearts at 48 hpf and 72 hpf were loaded on 10× Chromium Controller Chip G (10× Genomics) and processed according to the Chromium Next GEM Single Cell 3′ Reagent Kits v.3.1 targeting 10 000 cells. Resulting libraries were verified by High-Sensitivity DNA Kit (Agilent Technologies) on a 2100 Bioanalyzer (Agilent Technologies) and Qubit Fluorometer using Qubit dsDNA High-Sensitivity Assay Kit (Invitrogen). Final libraries were quantified with KAPA Library Quantification Kit Illumina Platforms (Kapa Biosystems/Roche), followed by paired-end sequencing (read 1 - 28 cycles, i7 index - 8 cycles, i5 index - 0 cycles, read 2 - 91 cycles) performed with Nextseq 500 (Illumina).

#### QC, filtering and clustering

Obtained raw sequencing data (BCL files) were demultiplexed and transformed into fastq files using 10× Cellranger pipeline version 3.1.0^127^ and bcl2fastq v2.19.0.316 (Illumina). The resulting matrices were loaded into the Seurat package v4.0.1^128^ for R v4.0.2^129^ applying standard quality control, normalization and analysis steps unless otherwise specified in the description. Briefly, sequencing reads were mapped to the zebrafish reference genome GRCz11 (Ensembl release 100) extended with additional EGFP and mRFP sequences. Due to overlapping annotations, reads mapped to non-polyA transcripts and protein-coding genes may be flagged as multi-mapped, and in consequence not count in the 10× pipeline, respective annotation GTF file was pre-filtered to retain only protein-coding genes (*cellranger mkgtf --attribute = gene_biotype:protein_coding*). The estimated number of cells identified by the Cellranger pipeline was 52,447 (Table S1). In order to dispose of potential empty droplets, low quality cells and possible multiplets, droplets with a very small (<200) and very high (>2500) library size were removed. Standard scRNA-seq filtering workflows exclude cells with a high ratio of reads from mitochondrial genome transcripts, which may indicate potential cell membrane rupture and dissociation-based damage. This filter is often set at 5–10%,^130^ however, the heart as muscle tissue is composed of diverse cell populations including cardiomyocytes which require high energy demand leading to potentially very high mitochondrial gene content per cell. Therefore, to retain cardiomyocytes while removing low-quality cells, we chose a threshold of 30% mitochondrial gene content. In addition to above mentioned thresholds, we also decided to remove cells containing more than 10% of hemoglobin reads. Altogether, to keep high quality cells the following thresholds were applied: 200 < number of genes <2500 & mitochondrial gene content <30% & hemoglobin gene content <10%. An increasing number of targeted cells is inherently associated with higher multiplet rate. To maximize the removal of possible doublets from the dataset, in addition to filtering out cells with very high gene content (>2500) we applied the DoubletFinder^131^ approach to exclude extra heterotypic doublets derived from transcriptionally distinct cells (Table S1). After applying all filtering steps, a total of 34 676 cells were retained for further analysis (Table S1). Following the Seurat workflow, the resulting gene expression matrices were normalized according to “SCT transform” using “glmGamPoi” method and the default number of cells and variable genes.^119^ The scaled matrices were dimensionally reduced by PCA and Uniform Manifold Approximation and Projection (UMAP) embedding followed by neighbor finding and clustering according to default Seurat workflow. In each, 30 principal components were used.

#### Integration of single-cell datasets

Having two biological pseudo-replicates for each timepoint (48 hpf and 72 hpf, respectively), data were integrated using the Seurat integration approach applying default parameters.^132^ 3 000 integrated anchors were identified and used as an input to create an integrated dataset as implemented in Seurat. Cells were clustered based on KNN graph based approach and Louvian algorithm for modularity optimization with the resolution parameter ranging from 0.5 to 12. Ultimately resolution was set to 1.5. Clustering results were visualized by UMAP. Finally, we used the built-in default Seurat *FindAllMarkers* and *FindMarkers* functions to identify differentially expressed genes in each cluster. As a background, the entire dataset was used except for differential expression analysis between myocardial subclusters that have been compared internally. Functional Gene Ontology (GO) analysis was performed online (http://geneontology.org/) using PANTHER classification system^133^ according to the default parameters (Fischer’s exact test and FDR as correction) or ClusterProfiler v. 3.17.3.^134^ Top 100 differentially upregulated genes were used as an input. However, many gene products involved in pacemaker development in zebrafish are not comprehensively captured by GO,^135^ therefore, the Ensembl IDs of all upregulated genes (adj. *p*-value <0.05) from Sinoatrial CMs cluster were converted to human orthologs and searched against *Homo sapiens.* All subsequent enrichment plots were generated in ggplot2 package using zebrafish ensembl IDs. The single cell transcriptome data can be accessed https://www.zfcardioscape.iimcb.gov.pl. Interactome map between the cardiac cell clusters was constructed using the DanioTalk tool.^57^ The tool provides zebrafish-focused annotations based on physical interaction data of the zebrafish proteome and hence provided significantly higher number of ligand-receptor interactions compared to others built on mammalian data. All sequencing data have been deposited in the GEO database under accession number GSE234216.

#### Spatial correlation analysis

The TOMO-seq dataset^31^ was downloaded and used as spatial reference. Genes that had a maximum read count of less than 20 were first removed. We then smooth the data with local regression method (LOESS, span ***α*** = 0.15) and calculate fold changes for all genes against the average for that gene across all sections. The Pearson correlation coefficient between each scRNA-seq clusters and each section is calculated. The spatial correlations (***ρ***) between the scRNA-seq clusters and the sections^31^ were calculated by applying the following steps: (1) Ensembl gene IDs were used to match the data from the clusters and the sections, (2) in the cluster data, the log2FC were converted to fold change values, (3) if resulting value for a given gene ID was zero either for clusters or sections, these gene ID was ignored, (4) the Pearson correlation coefficient ***ρ*** (*x*) between the cluster and each section *x* was estimated. The average number of compared genes for all sections was 254. As a proxy for assessing quality of comparison, we investigated the number of genes that were compared for estimation of ***ρ*** for each section with respect to this average value. The sections from 2nd to 36th had number of compared genes between 0.8 and 1.2 of the average value, and only sections 37th to 39th had 0.2 of the average value. Section IDs are as in Burkhard and Bakkers.^31^

#### Zebrafish F0 knockout of candidate genes using CRISPR/Cas9

The sgRNA sequences were designed utilizing chopchop^136^ with default settings for CRISPR/Cas9 knock-out method. Based on previously established protocol,^71^ sgRNAs were designed to target three different loci per gene. The following exons were targeted: 2nd, 4th and 5th as well as 2nd and 6th (in two *loci*) for *atp1b3b* and *colec10*, respectively (Figure 4B; Table S2). All selected sgRNAs were specific to the targeted gene showing no mismatches and off-targets according to chopchop (Table S2). Synthetic sgRNAs were ordered from Synthego at 1.5 nmol scales and dissolved in 15 μL of 1× TE buffer (10 mM Tris-Hcl, 1 mM EDTA, pH = 8) to reach 100 μM stock concentration. Sequences and all quality metrics are provided in Table S2. Cas9 protein was ordered from IDT (Alt-R S.p. Cas9 Nuclease V3, catalog number 1081059). Based on,^137^ a pre-assembled complex made up of Cas9 and gRNA results in greater efficiency than the coinjection of Cas9 and gRNA. Therefore, one day prior to scheduled injections, individual sgRNAs were mixed in equal volumes and adjusted to 19 μM sgRNA mix concentration. sgRNA mixes were combined with previously diluted Cas9 protein in Cas9 buffer (20 mM Tris-HCl, 600 mM KCl, 20% glycerol^70^) to reach a final concentration of 9.5 μM RNP complex concentration (molar ratio 2:1, 19 μM sgRNA mix: 9.5 μM Cas9) and incubated at 37°C for 5 min then cooled on ice and stored in 4°C until microinjection. Approximately 1 nL of RNP complex composed of three sgRNA targeting different gene loci and Cas9 protein was injected into the yolk of one-cell stage embryos.^70^ Uninjected siblings were used as control. In the case of both *atp1b3b* and *colec10* knockdown, injection of 28.5 μM of Cas9-sgRNA complex according to previous report^71^ induced over 70% mortality which suggests batch-sepcific toxicity of the Cas9 enzyme (not shown), therefore we used 9.5 μM in our experiments. To facilitate the observation of the heart, all experiments were performed in the transgenic line *Tg(myl7:EGFP)*^121^ or *Tg(myl7:EGFP-Hsa.HRAS)*^*s883*^^122^ expressing EGFP in the heart. To account for possible off-target effects resulting from microinjections, control embryos were injected with scrambled RNP complex composed of Cas9 protein and gRNA (same as in Kroll et al.^71^; molar ratio 2:1, 19 μM sgRNA mix: 9.5 μM Cas9) that does not have a complementary sequence in the genome to direct a Cas9-mediated genome cleavage (Table S2).

#### Knockout phenotype and heart rate analysis

Following microinjection, dead embryos before 24 hpf were discarded to exclude unfertilized embryos or those damaged by the injection. Phenotypic analysis was performed starting from 48 hpf. First, malformed embryos and/or embryos that developed cardiac edema were separated and reported. Then, wherever possible, a random 10 and 20 embryos from the uninjected control and knockout groups were sampled for heart rate analysis and kept for further observations. Heart rate analysis was performed at 48 hpf on the SZX16 fluorescent stereomicroscope (Olympus) by manually counting the number of heartbeats for 30 s per embryo using a timer. To maximize the accuracy of the heart rate measurements at a later stage (72 hpf), fluorescent hearts of randomly selected individuals from both, uninjected siblings and knockout embryos were recorded for 10 s (34 fps), followed by heart rate analysis using pyHeart4fish software (fluorescent mode, 34 fps).^138^ Subsequent statistical analysis was performed in R. Shapiro-Wilk normality test implied that the distribution of the knockout data are significantly different from a normal distribution (*p*-value <0.05), therefore, non-parametric Wilcoxon rank-sum test was applied to compare data between wildtype and knockout embryos. The Holm-Bonferroni method was used for adjusting *p*-values. All movies were recorded using acA1440-220 μm USB 3.0 camera (Basler).

#### Identification of CRISPR/Cas9-induced indels

Genomic DNA was isolated from a single whole embryo in its chorion at 24 hpf according to the HotSHOT method.^139^ In brief, an individual embryo was transferred to a PCR tube containing 50 μL of 50 mM NaOH, followed by 10 min incubation at 95°C and ice-cooling. The solution was then neutralized by the addition of 1/10 volume of 1 M Tris–HCl pH 8 and mixed. The sample was centrifuged to pellet the debris. Diluted supernatant was used for high-resolution melting (HRM) analysis.^140^ PCR primers were designed using the NCBI Primer-BLAST^141^ to amplify genomic regions flanking the Cas9-nuclease cleavage sites (Figures S5C and S5D; Table S3). Due to difficulties in the amplification of regions of interest, a custom HRM mix was utilized. The PCR reactions were made with 0.2 μL Phire Hot Start II DNA Polymerase (Thermo #F122S), 0.5 μL of each primer (10 μM), 1 μL of previously diluted genomic DNA, 0.6 μL of SYBR Green I dye and water up to 10 μL. Depending on the variant, the PCR reaction was supplemented with DMSO ranging from 3 to 6%. The HRM was performed in a LightCycler 480 Instrument II (Roche) using white 96 well plates and a default curve melting program. Melting curves were analyzed using the Roche LightCycler 480 software version 1.01.01.0050. To improve readability, melting curves were recreated in R using the ggplot2 package.^142^

#### Spatial gene expression validation by *in situ* hybridization

Whole mount *in situ* hybridization was performed as previously described.^33^ The following primers were used for probe synthesis: 5′-CTCATTGCCTCCACAACGCC-3’ (forward primer) and 5′-AGAGGAACTGACATCGTGCG-3’ (reverse primer) which 5′-end was extended with the following T7 polymerase promoter sequence: 5′-TAATAC GACTCACTATAGGGAGA-3’. Probes and reagents required for hybridization chain reaction *in situ* (HCR)^143^ were designed by and ordered from Molecular Instruments. HCR *in situ* was performed at 48 hpf and 72 hpf embryos according to the HCR RNA-FISH v3.0 manufacturer’s protocol optimized for zebrafish (Molecular Instruments). Larvae were treated with 10 μg/mL proteinase K (Roche) for 30 min and 50 min for 48 hpf and 72 hpf, respectively. To acquire images, embryos were mounted in 1% low-melting agarose and imaged using a Zeiss LSM 800 inverted confocal microscope.

### Quantification and statistical analysis

Single-cell RNAseq data analysis utilized statistical methods incorporated into the Seurat package. Statistical tests applied for spatial correlation analysis were extensively described in the applicable method section. Wilcoxon rank-sum test was applied for the heartbeat rate analysis.

## Supplemental information

Document S1. Figures S1–S6

Table S1. Single cell sample statistics for each biological replicate, related to Figure 1 and S1

Table S2. CRISPR sgRNA sequences, related to Figure 4 and S6

Table S3. High resolution melting curve analysis primer sequences, related to Figure 4 and S5

Table S4. Number of cells in each main cardiac cell cluster, related to Figure 1 and S1

Table S5. Marker genes expressed in each main cardiac cell cluster, related to Figure 1 and S1

Table S6. Spatial correlation between main and fine-grained clusters, related to Figure 2 and S2

Table S7. Cellular crosstalk between cell clusters based on ligand-receptor expression, related to Figure 2

Table S8. Enriched GO terms in myocardial cluster, related to Figure 3 and S3

Table S9. Marker genes expression in myocardial subclusters, related to Figure 3 and S3

Table S10. Enriched GO terms in “Sinoatrial CMs” subcluster, related to Figure 3 and S3

Table S11. List of genes encoding transcription factors, ion channels, and cell adhesion molecules in myocardial subclusters, related to Figure 3 and S3

Table S12. Myocardial cluster genes associated with ClinVar heart conditions, related to Figure 3 and S3

Table S13. Heartbeat rate measurements in CRISPR knockout experiment, related to Figure 4 and S6

Table S14. Marker genes expression in endothelial subclusters, related to Figure 5

Table S15. Enriched GO terms in endothelial subclusters, related to Figure 5

Table S16. Marker genes expression in neural subclusters, related to Figure 5

Table S17. Enriched GO terms in neural subclusters, related to Figure 5
