# Supplementary material for: scRNA-seq reveals the diversity of the developing cardiac cell lineage and molecular players in heart rhythm regulation
Source: iScience. 2024 May 22;27(6):110083. doi: 10.1016/j.isci.2024.110083 (PMC11170199; doi:10.1016/j.isci.2024.110083)
Supplement: Document S1. Figures S1–S6 [file mmc1.pdf]

iScience, Volume *27*

Supplemental information

scRNA-seq reveals the diversity of the developing cardiac cell lineage and molecular players in heart rhythm regulation

Karim Abu Nahia, Agata Sulej, Maciej Migdał, Natalia Ochocka, Richard Ho, Bożena Kamińska, Marcin Zagorski, and Cecilia Lanny Winata

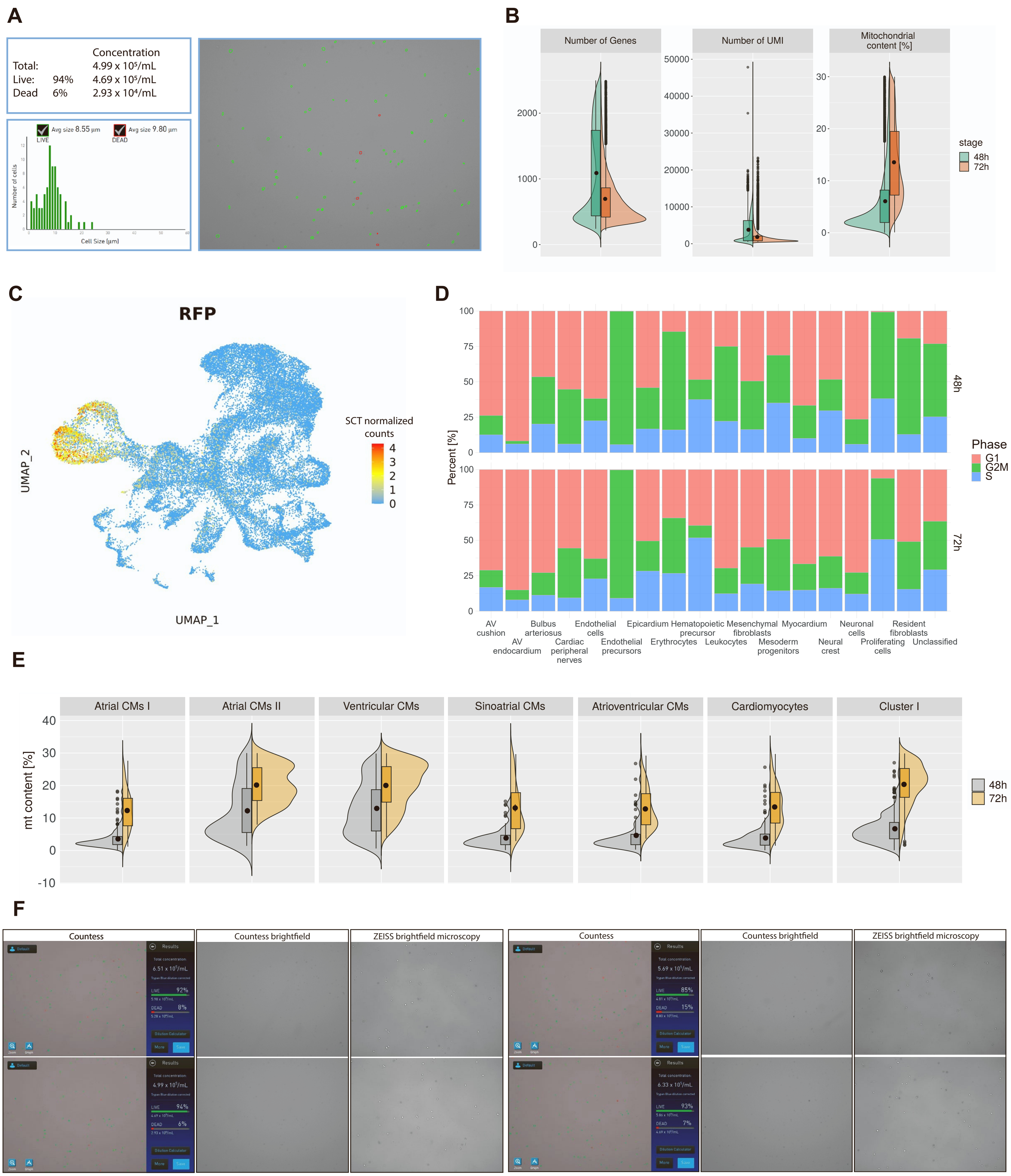

Supplementary Figure 1. Quality parameters of scRNA-seq dataset. A Example of the processed image showing quality and quantity check of single cell suspension by automated cell counter. Viable cells are marked as green. For raw data refer to **F**. **B** Violin plot showing mitochondrial gene content, number of expressed genes, and UMI at each developmental stage. **C** UMAP plot of cardiac cell clusters indicating the cardiomyocyte expression of RFP reporter gene (SCT normalized counts). **D** Composition of cells in each main clusters expressed as a percentage according to their cell cycle phase. **E** Mitochondrial gene content in each myocardial cluster depending on developmental stage. **F** Raw images of heart dissociation metrics obtained by Countess Automated Cell Counter showing typical yield and quality of obtained cell suspension. To further substantiate the successful cell dissociation, each automated measurement (Countess) is supported by ZEISS brightfield microscopy taken each time before proceeding with cell encapsulation. Related to Figure 1, Table S1, S4, and S5.

A

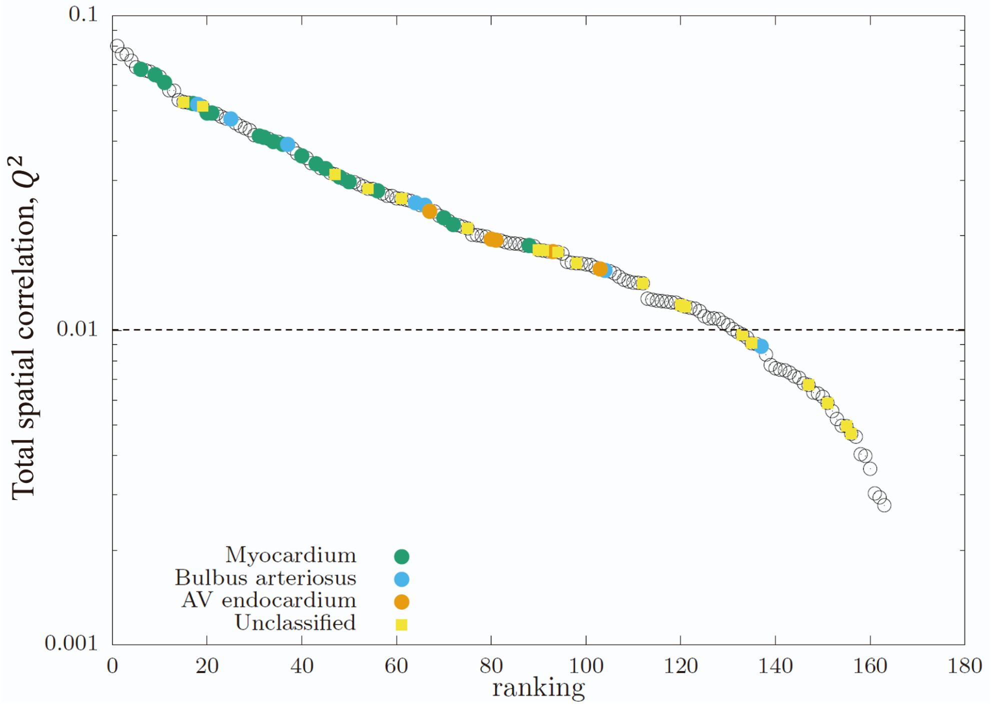

Cluster	Q^2
Bulbus arteriosus	0,1070
Erythrocytes	0,0830
Mesoderm progenitors	0,0760
Resident fibroblasts	0,0610
Proliferating cells	0,0520
Epicardium	0,0450
Hematopoietic precursor	0,0350
Endothelial precursors	0,0300
Myocardium	0,0300
AV endocardium	0,0210
Endothelial cells	0,0200
Leukocytes	0,0190
AV cushion	0,0180
Neuronal cells	0,0160
Cardiac peripheral nerves	0,0150
Neural crest	0,0130
Mesenchymal fibroblasts	0,0130
Unclassified	0,0050

B

Cluster	Composition
115	99.29% Mesoderm progenitors, 0.71% Bulbus arteriosus
26	100% Mesoderm progenitors
31	95.21% Erythrocytes, 4.79% Unclassified
28	100% Erythrocytes
5	86.67% Bulbus arteriosus, 2.86% Epicardium, 10.48% Unclassified

Supplementary Figure 2. Correlations between main and fine-grained clusters. A Spatial correlation between main cluster and 164 fine-grained clusters. Q^2 represents the sum of the square of the Pearson correlation coefficient from all sections normalised by the number of sections. The Q^2 for main clusters is given in the table. The Q^2 for fine-grained clusters was ranked from the highest to the lowest. The highlighted fine-grained clusters are associated with one of the selected main cluster ("Myocardium", "Bulbus arteriosus", "AV endocardium", and "Unclassified"), if 90% of points in that cluster belong to the respective main cluster. The "Myocardium" fine-grained clusters are quite high with "Bulbus arteriosus" fine-grained clusters ranked slightly lower. The dashed line indicates roughly the maximum level of Q^2 obtained by calculating spatial correlation with randomized gene order for several randomly selected fine-grained clusters. **B** Top 5 fine-grained clusters with the highest Q^2. Related to Figure 2 and Table S6.

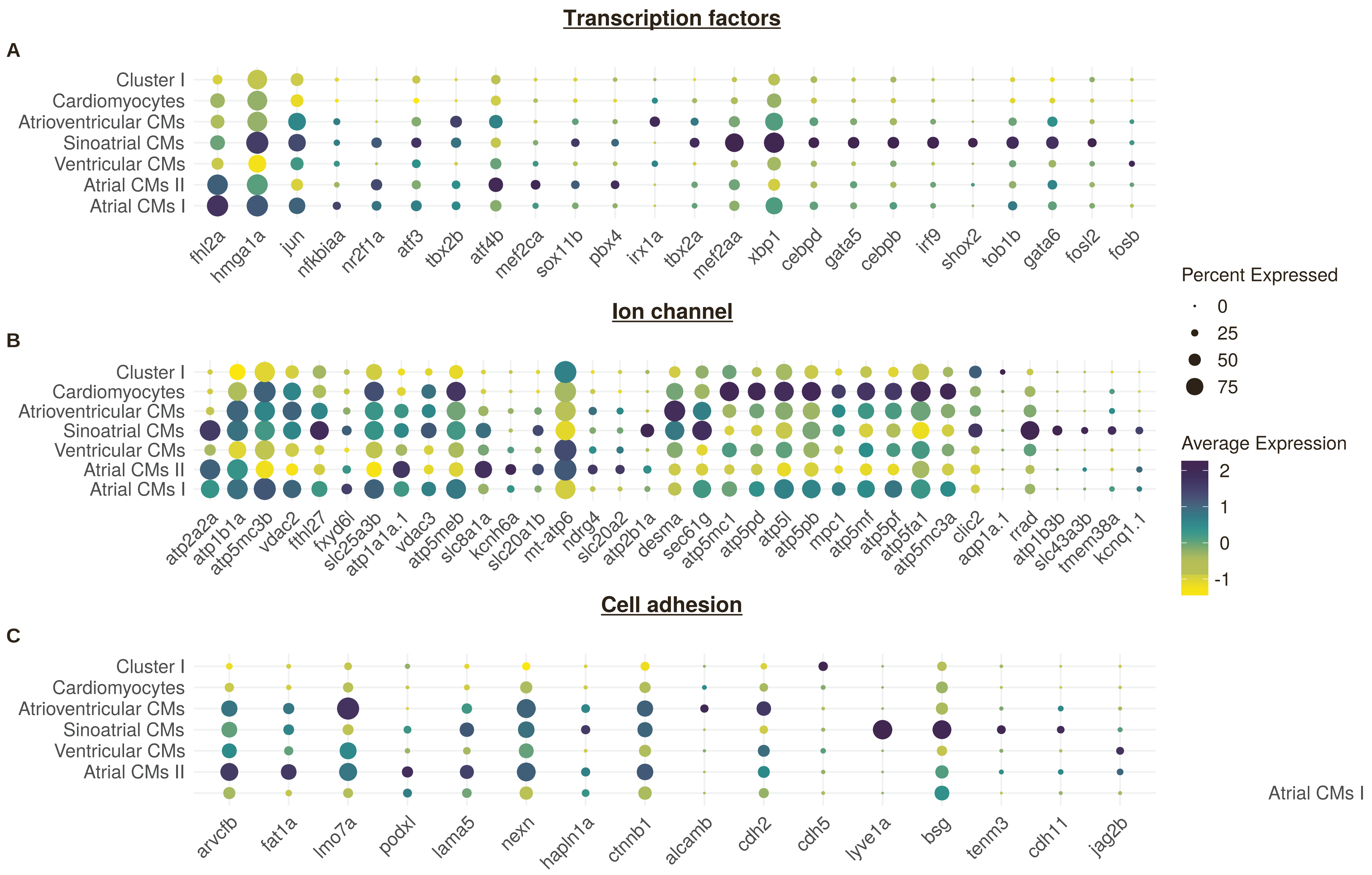

Supplementary Figure 3. Overview of upregulated genes related to the transcription factor (A), ion channel (B), and cell adhesion (C) activity among cardiomyocyte clusters. Related to Figure 3, Table S8-12.

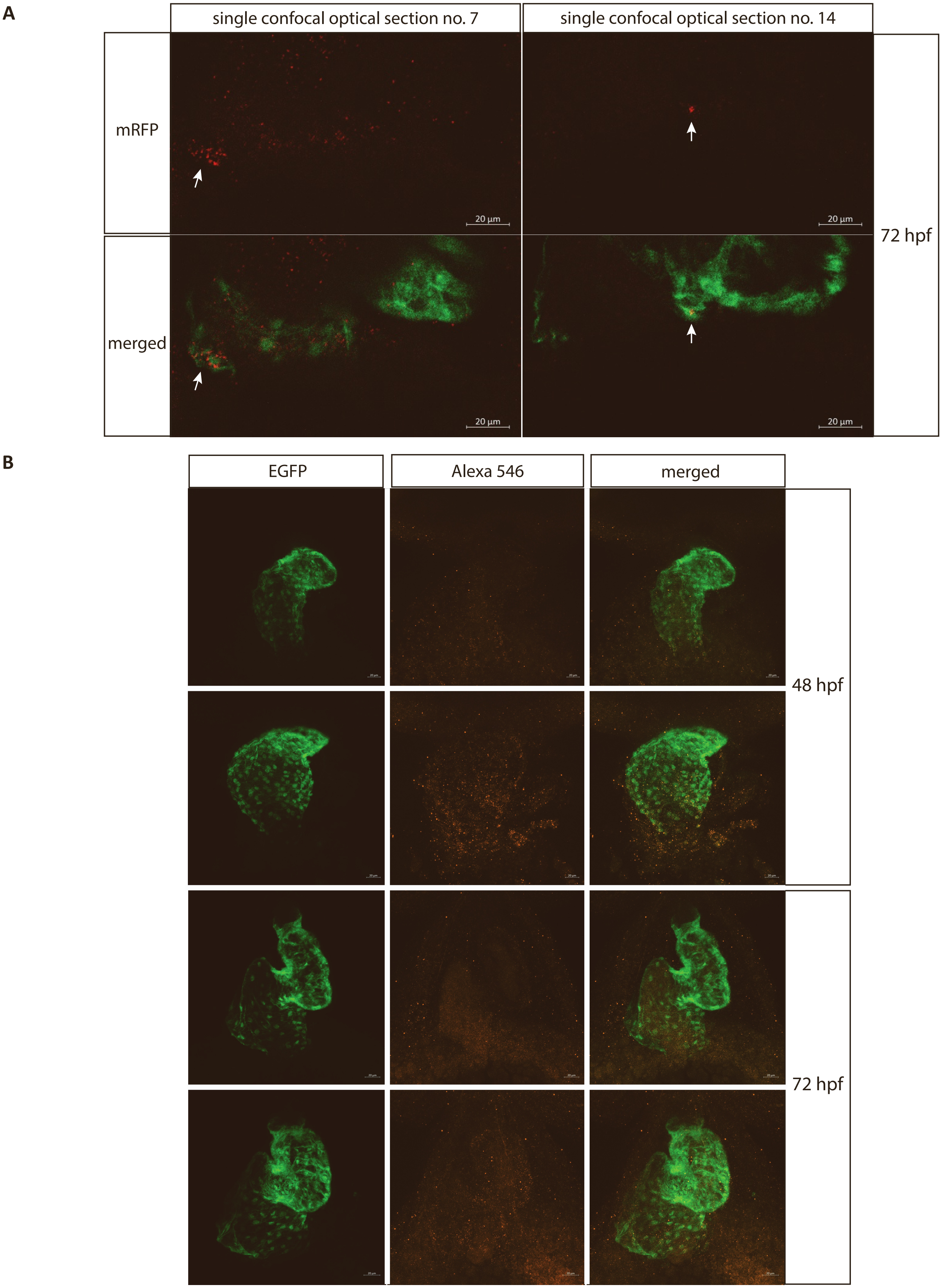

Supplementary Figure 4. The hybridization chain reation (HCR) *in situ* for *atp1b3b* (A) and *colec10* (B) was performed on 48 hpf and 72 hpf larvae. (A) In reference to Fig. 4 H, single confocal optical sections of *atp1b3b* HCR *in situ* performed on 3 dpf larvae. The internal EGFP expression on *Tg(myl7:EGFP)* labels cardiomyocytes in green. *Atp1b3b* and *colec10* expression is visualized by a dedicated HCR probe and hairpins conjugated with Alexa-647 and Alexa-546 fluorophores, respectively. In both developmental stages, no clear *colec10* expression was detected **(B)**. Related to Figure 4.

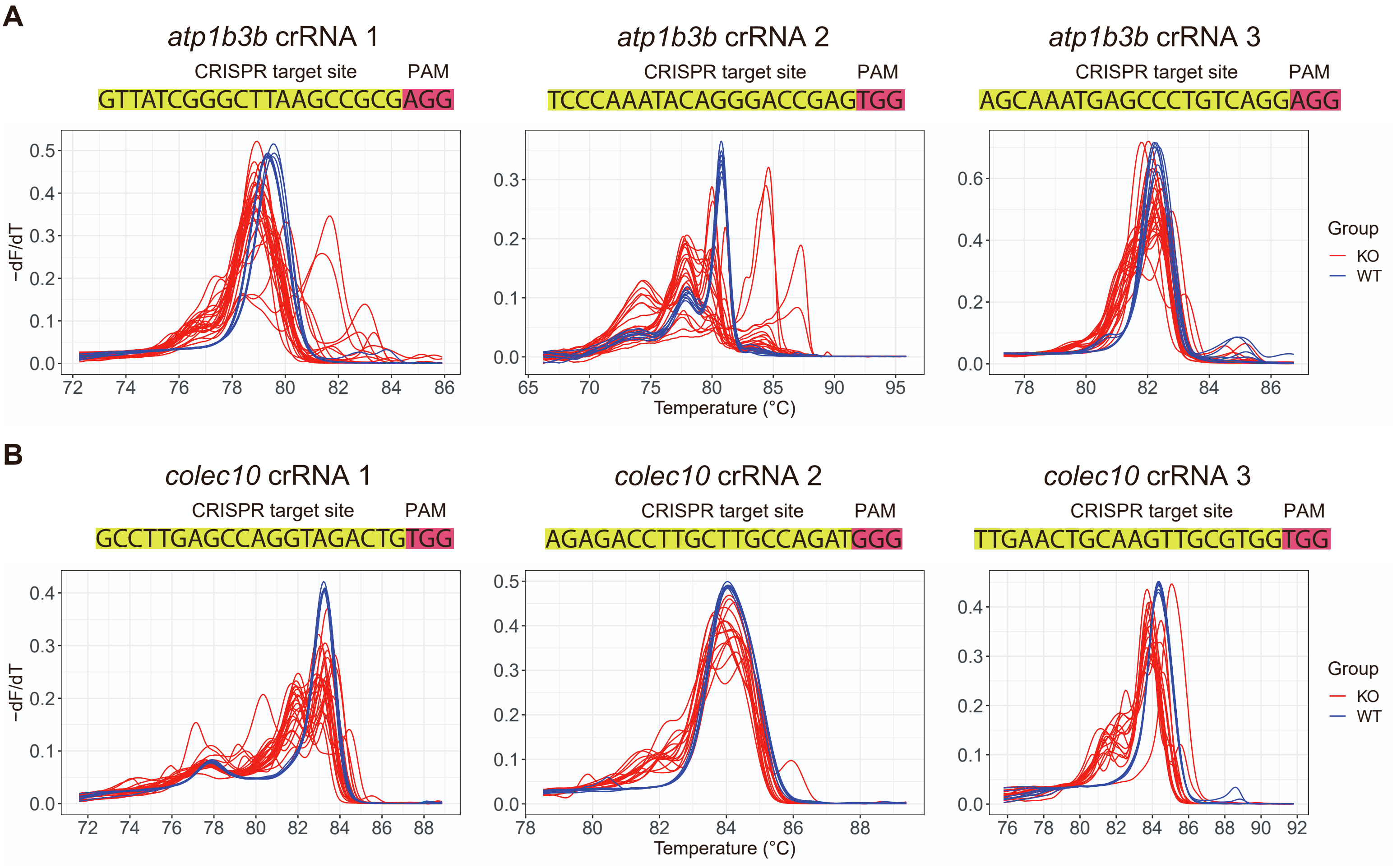

C

atp1b3b			***colec10***		
crRNA 1	crRNA 2	crRNA 3	crRNA 1	crRNA 2	crRNA 3
24/24 (100%)	20/22 (91%)	19/24 (79%)	24/24 (100%)	21/23 (91%)	23/24 (96%)
178 bp	162 bp	264 bp	208 bp	247 bp	175 bp

D

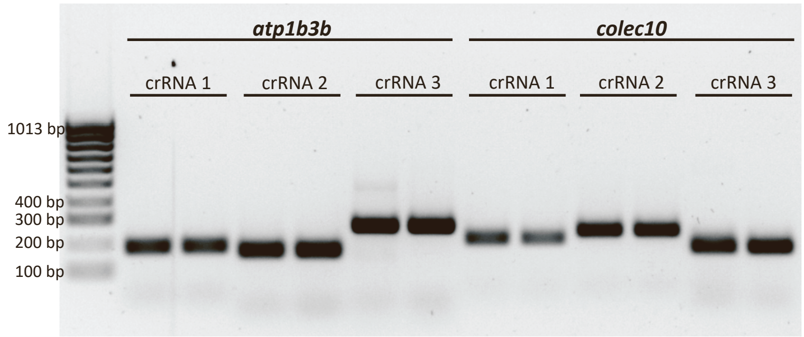

Supplementary Figure 5. High-resolution melting (HRM) analysis was used to validate the efficacy of individual crRNAs for each gene. The melting curves of 22-24 independent embryos injected with the RNP complex containing either *atp1b3b* **(A)** or *colec10* **(B)** crRNA1, crRNA2 or crRNA3 are presented (red). The melting curves of 8 independent uninjected sibling embryos are also presented for comparison for each crRNA (blue). Injection of each RNP complex resulted in the disruption of the corresponding target genomic sequences. **(C)** Table summarizing the efficiency of each crRNA-mediated gene loci disruption**.** **(D)** Products of amplification of genomic DNA from uninjected 24 hpf embryos encompassing the crRNA target regions for *atp1b3b* and *colec10* resolved in gel electrophoresis. Related to Figure 4, Table S3.

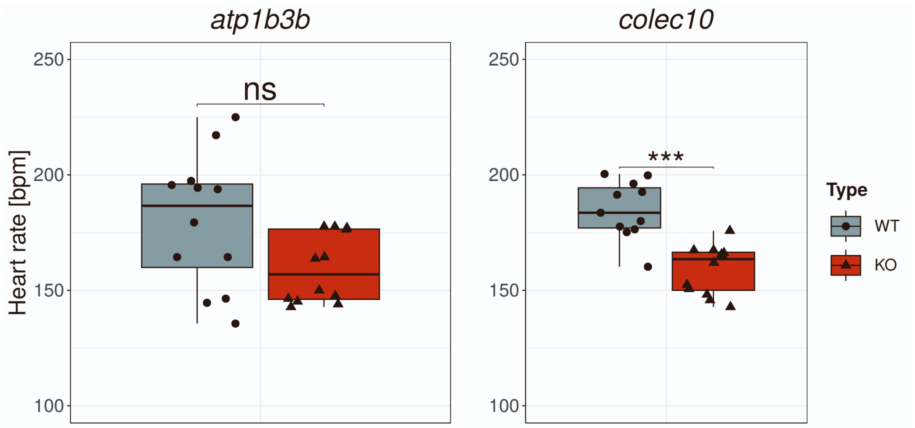

Supplementary Figure 6. Boxplot showing differences in heart rate between each targeted gene knockout embryos and corresponding uninjected control siblings at 72 hpf. Each point represents heart rate of individual embryo (n = 12, from two independent experiments). Heart rate data were acquired by 10s long video recording (34 fps) and then subjected to detailed analysis utilizing pyHeart4fish software (Vedder et al. 2023). Statistical test: Wilcoxon rank sum test. Related to Figure 4, Table S2, S13, Movie S1 and S2.
